# Females with Fabry disease: an expert opinion on diagnosis, clinical management, current challenges and unmet needs

**DOI:** 10.3389/fcvm.2025.1536114

**Published:** 2025-03-12

**Authors:** Antonino Tuttolomondo, Cristina Chimenti, Vittoria Cianci, Maurizio Gallieni, Chiara Lanzillo, Antonella La Russa, Giuseppe Limongelli, Renzo Mignani, Iacopo Olivotto, Federico Pieruzzi, Antonio Pisani

**Affiliations:** ^1^ Department of Internal Medicine and Stroke Care, University Policlinico Hospital of Palermo, and ProMISE Department, University of Palermo, Palermo, Italy; ^2^ Department of Clinical, Internal, Anesthetic and Cardiovascular Sciences, La Sapienza University of Rome, Rome, Italy; ^3^ Neurology and Stroke Care Unit, Great Metropolitan Hospital, Bianchi-Melacrino Morelli, Reggio Calabria, Italy; ^4^ Dipartimento di Scienze Biomediche e Cliniche, University of Milano, Milano, Italy; ^5^ Division of Cardiology, Policlinico Casilino, Rome, Italy; ^6^ Department of Health Sciences, University of Magna Graecia, Catanzaro, Italy; ^7^ Department of Translational Medical Sciences, AORN dei Colli—University of Campania Luigi Vanvitelli, Naples, Italy; ^8^ Department of Medical and Surgical Sciences (DIMEC), Alma Mater Studiorum University of Bologna, Bologna, Italy; ^9^ Nephrology Department, IRCCS S. Orsola Hospital, University of Bologna, Bologna, Italy; ^10^ Pediatric Cardiology, Meyer Children’s Hospital IRCCS, Florence, Italy; ^11^ Nephrology, Fondazione IRCCS San Gerardo dei Tintori, Monza, Italy; ^12^ School of Medicine and Surgery, University of Milano-Bicocca, Milan, Italy; ^13^ Department of Public Health, Federico II University of Naples, Naples, Italy

**Keywords:** alpha-galactosidase A, enzyme replacement therapy, Fabry disease, female, genetic testing, heterozygote

## Abstract

Females with Fabry disease (FD) often have a milder phenotype, later symptom onset, and slower disease progression than males, causing delayed diagnosis and undertreatment. A survey was conducted at nine Italian FD centers to evaluate routine management of females with FD; results were discussed at a meeting of eleven Italian specialists and recommendations developed. Of the 227 females managed by the physicians surveyed, 85% were diagnosed through family screening and 38.5% were symptomatic at presentation. Female patients usually underwent cardiac, renal, and neurologic monitoring, and measurement of plasma lyso-globotriaosylsphingosine (Gb3) levels at 6- or 12-month intervals. Treatment was initiated in 54%, mostly enzyme replacement therapy. Experts recommended screening all female relatives of index cases and evaluating all potentially affected organ systems. Diagnosis should be based on genetic analysis. Individualized monitoring of asymptomatic females must balance the need to detect organ damage while maintaining adherence. Treatment decisions should be based primarily on signs/symptoms of FD, but age, family screening results, *GLA* mutations, Gb3/lyso-Gb3 accumulation, and organ damage should be considered in asymptomatic females. More research on FD in females is needed and physicians should be aware of differences in the diagnosis, monitoring, and management of females vs. males with FD.

## 1 Introduction

Fabry disease (FD) is a rare X-linked lysosomal storage disorder caused by the lack or absence of lysosomal alpha-galactosidase A (α-Gal A) activity secondary to mutations in the α-Gal A gene (*GLA*) at Xq22.1 (1). This deficiency in α-Gal A leads to the progressive accumulation of its substrate globotriaosylceramide (Gb3) and its deacylated form lyso-Gb3, resulting in a multisystemic disease that mainly affects the kidneys, heart, and nervous system (1–3).

FD is often categorized into the classical phenotype, which develops in early childhood, is marked by absent or severely reduced α-Gal A activity, and has a severe outcome, and the non-classical phenotype, characterized by residual enzyme activity, variable disease course, age of onset, and manifestations, and has less severe outcomes (2, 4). Genomic screening has led to the identification of pathogenic and non-pathogenic variants, as well as *GLA* variants of unknown significance (VUS) (5).

The phenotype varies depending on the *GLA* variant, residual α-Gal A activity, age, gender, and, in heterozygous females, X chromosome inactivation (XCI) (5, 6). This phenotypic heterogeneity complicates FD diagnosis, particularly when a VUS is present (5). The increasing number of these variants highlights the need to clarify the pathogenic role of VUS when making treatment decisions.

The X-linked inheritance of FD means that hemizygous males are typically more severely and consistently affected, whereas heterozygous females present a variety of symptoms, ranging from asymptomatic to severe (4). Overall, females with FD tend to have a milder phenotype, later onset of symptoms, and slower disease progression than males (4, 7). This variability in females depends on the pathogenic variant and the XCI profile (8, 9). XCI is random, dependent on the cell type, and frequently non-uniform across the silenced chromosome (10), leading to different patterns of Gb3 accumulation.

As a multisystemic disease, the diagnosis, monitoring, and management of FD should involve a multidisciplinary clinical team, including an internist, neurologist, nephrologist, cardiologist, medical geneticist, genetic counsellor, psychologist, and nurse. At present, four therapies are approved in Europe for FD: three enzyme replacement therapies [ERTs; i.e., agalsidase alpha (agalsidase-α, Replagal®, Takeda UK, Ltd), agalsidase beta (agalsidase-β, Fabrazyme®, Sanofi), and pegunigalsidase-α (Elfabrio®, Chiesi Farmaceutici S.p.A.)], and one pharmacologic chaperone [i.e., migalastat (GalafoldTM, Amicus Therapeutics)].

There are many challenges and unmet needs in the diagnosis and clinical management of females with FD. FD diagnosis is often missed in females, who are still sometimes considered as mere carriers of a defective *GLA* gene, particularly when paucisymptomatic (11). Thus, many female patients remain undiagnosed for more than 10 years after they first experience symptoms (12, 13). Furthermore, there is evidence that female patients are undertreated in spite of having major organ involvement, such as left ventricular hypertrophy (LVH) and stage 3 chronic kidney disease (CKD) (13–16). Moreover, recommendations from an international panel of FD specialists specify that males with classical FD should receive treatment regardless of symptoms, whereas females with classical FD should only receive treatment following the appearance of major organ injury (17). Thus, the appropriate timing of treatment in female FD patients remains an unanswered question, as does the optimal follow-up and monitoring strategy.

An Italian advisory board meeting of expert clinicians was held on 7 July 2022 to discuss the challenges and unmet needs in the diagnosis and clinical management of females with FD based on the results of a survey conducted at nine Italian FD centers. The survey was conducted to understand how female patients with FD are treated in clinical practice today and to compare real-life approaches with what is documented in the literature. The purpose of the meeting was to highlight the specific features of FD in females pertaining to presentation and disease progression, and the consequent diagnostic, therapeutic, and follow-up requirements, aiming to promoting greater awareness and better management of female patients with FD. The main conclusions of the meeting are summarized here.

## 2 Survey questionnaire and advisory board meeting

Eleven Italian clinicians (four cardiologists, one neurologist, one internist/neurologist, four nephrologists, and one geneticist) provided their expert opinion, based on the survey results, and shared their clinical experiences; these clinicians are the authors of this article. The advisory board meeting was sponsored by Sanofi.

The survey questionnaire was designed by the Sanofi medical team based on the topics to discuss at the meeting (clinical presentation, and disease progression and monitoring). The survey questionnaire was reviewed Dr Mignani and Prof. Tuttolomondo and then emailed to the survey participants between 20 and 28 June 2022. Physicians at participating centers were asked to provide information about the characteristics and management of female patients with FD at their center. The questionnaire included nine items regarding the screening and diagnosis of female patients, disease characteristics, type and frequency of disease monitoring after FD diagnosis, factors considered for treatment initiation, and their treatment status (Supplementary Material). Nine of 11 participating Italian FD centers responded to the survey and provided data on 227 female patients with FD who were being treated or monitored if untreated. The survey sample included both symptomatic and asymptomatic cases of females with FD, as well as patients whose symptoms were initially not recognized as related to FD. Data were collected and analyzed by the Sanofi medical team in collaboration with Prof. Tuttolomondo.

## 3 Survey findings and analysis

### 3.1 Genetic and biochemical aspects of FD in females and diagnostic implications

#### 3.1.1 Survey findings

Among the 227 female patients with FD in the survey, 85% were diagnosed through family screening and diagnosis was based on clinical manifestations in the other 15%. All centers used molecular genetic testing for the identification of *GLA* mutations encoding an absent or evidently dysfunctional α-Gal A. While many centers perform enzymatic assays for α-Gal A activity quantification and measure Gb3 or lyso-Gb3 concentrations, none of the centers rely on these parameters for the diagnosis of FD in females. Instead, these parameters are used to inform treatment decisions. XCI evaluation, as a predictive marker of disease progression, was not performed in any of the centers.

#### 3.1.2 Analysis

It is well known that male patients with FD can be diagnosed by α-Gal A activity testing alone, but in female patients, it is necessary to demonstrate a disease-causing mutation in the *GLA* gene as the plasma α-Gal A activity is usually normal or highly variable due to skewed XCI (9). Genetic diagnosis in females with FD can be even more challenging in the case of deletions or duplications, usually associated with severe phenotype, as this type of mutation cannot be assessed by direct DNA sequencing but only by specific techniques such as multiplex ligation-dependent probe amplification (MLPA) (18).

In males with FD disease where there is only one X chromosome, genetic diagnosis cannot be missed. However, in females with two X chromosome copies (one normal and the other affected in different proportions in different tissues), these types of mutation (deletions and duplications) cannot be detected with routine genetic tests (19). Heterozygosity of the X chromosome in females makes it important to combine routine sequencing analysis with allelic dosage assays, such as MLPA, to more reliably exclude or confirm FD (18).

FD diagnosis based on lyso-Gb3 levels is sometimes unreliable, as lyso-Gb3 levels vary depending on the disease phenotype, increasing significantly in classical FD but increasing slightly in non-classical FD, while in VUS, lyso-Gb3 level at diagnosis is quite variable but mostly normal (20, 21). Therefore, lyso-Gb3 levels can be a useful tool for the diagnosis of classical vs. non-classical FD in females (20, 22). Moreover, baseline lyso-Gb3 level predicts disease severity over time and is associated with important clinical events (23). Lyso-Gb3 levels can also be useful to assess treatment response in females as it usually decreases upon initiation of ERT or chaperone therapy (23).

Although this survey reported no epigenetic evaluation, the XCI profile may guide treatment decisions in female patients (17). XCI in females is an epigenetic mechanism occurring during embryonic development to ensure X-linked dosage compensation between cells of females (XX karyotype) and males (XY) (24). During XCI, one of the X chromosomes in each cell is randomly silenced and converted into a Barr body, resulting in the mosaic expression of X-linked genes in different organs/tissues. This is facilitated by X-inactive specific transcript (Xist), a non-coding RNA, which coats its chromosome of origin, recruits heterochromatin factors and silences gene expression.

An estimated 15%–30% of the genes within Barr bodies escape inactivation in a constitutive (10%) or variable (90%) manner, leading to biallelism, which may be beneficial for females (25). The maternal vs. paternal X-inactivation ratio is generally 50:50, but imbalances may occur due to genetic mechanisms, such as XIST gene mutations. X chromosome imbalance increases with age in all women (26), leading to worsening disease manifestations in those with FD (9). Analysis of XCI imbalance in different tissues previously showed a correlation between the XCI patterns of blood and other tissues, and significant differences in residual α-Gal A levels, severity scores, progression of cardiomyopathy, and deterioration of kidney function depending on the direction (random XCI, wild-type allele or mutant allele expression) and degree of XCI imbalance (9). This led to the conclusion that XCI significantly impacts the phenotype and natural history of FD in females, and that monitoring and therapeutic intervention depends on the predominantly expressed allele (i.e., wild-type GLA expression leads to mild phenotype and minimal disease progression, whereas mutant GLA expression leads to severe phenotype, rapid progression with age, and poorer prognosis) (9).

Because XCI influences FD disease severity, its early characterization may help identify asymptomatic patients at risk of developing severe disease and requiring early medical attention (27). However, in contrast with previous studies, a 2021 meta-analysis showed no correlation between XCI imbalance and phenotype in female FD carriers (19).

The HUMan Androgen Receptor gene Assay (HUMARA) test is the most widely used method for analyzing XCI imbalance (28). However, because HUMARA only tests a single locus, methylation status of only a few CpG islands are assessed; therefore, it does not reflect which allele has a pathogenic variant and it cannot predict clinical severity as it does not evaluate mRNA expression (29).

More recently, ultra-deep targeted RNA sequencing has been introduced, using next-generation sequencing (NGS) to examine locus-specific methylation within a single cell of a tissue (30). The benefits of NGS include detection of all the CpG islands and the degree of methylation and expression of genes at any given time, and the direct measurement of mutated-to-wild-type alleles ratio in the *GLA* gene at the mRNA level, and consequently, the influence of XCI on clinical manifestations (29). However, these data on epigenetic patterns data need to be statistically validated (31).

Methylation-based assessments are still not widely used in Italy; most centers perform the HUMARA test because it is simple and cost-effective. Despite being complex, ultra-deep RNA sequencing with NGS can be simplified for creating family trios and will be relatively affordable if performed on selected cohorts of patients. However, at present, only *GLA* gene sequencing using the Sanger method is being performed. The experts agreed that there was a lack of reliable evidence linking genotype and phenotype in females with FD and stressed the need for a reliable molecular method based on available evidence.

A direct method for XCI characterization involves family trio-based integrated whole-exome and mRNA sequencing, which can identify potentially pathogenic genetic mutations and XCI ratio using phased and unphased allele-specific expression analysis (32). Working with family trios can reduce genetic variability, improve the evaluation of genotype-phenotype correlation, and facilitate biomarker detection (32).

Extensive research has been conducted to identify novel markers that can potentially be used as screening/diagnostic tools and for the assessment of treatment response. An ideal biomarker should be easily evaluable, reliable, reproducible, organ-specific, able to accurately diagnose FD and assess disease expression early (before organ damage begins), and characterized by prognostic power (33). Clinical assessment requires the selection of appropriate tissue for testing (34); for example, podocyte testing and blood testing will generate different results. Podocytes can be examined by studying urinary extracellular vesicles, which are easily accessible and contain genetic material that is an informative source of cell-cell interactions and epigenetic modifications in renal cells (35). Exosomes within other bodily fluids may allow similar assessments of changes in the heart or other tissues that are not directly accessible, meaning that blood is no longer the only tissue studied.

### 3.2 Clinical manifestations and disease monitoring

#### 3.2.1 Survey findings

**Figure 1:**
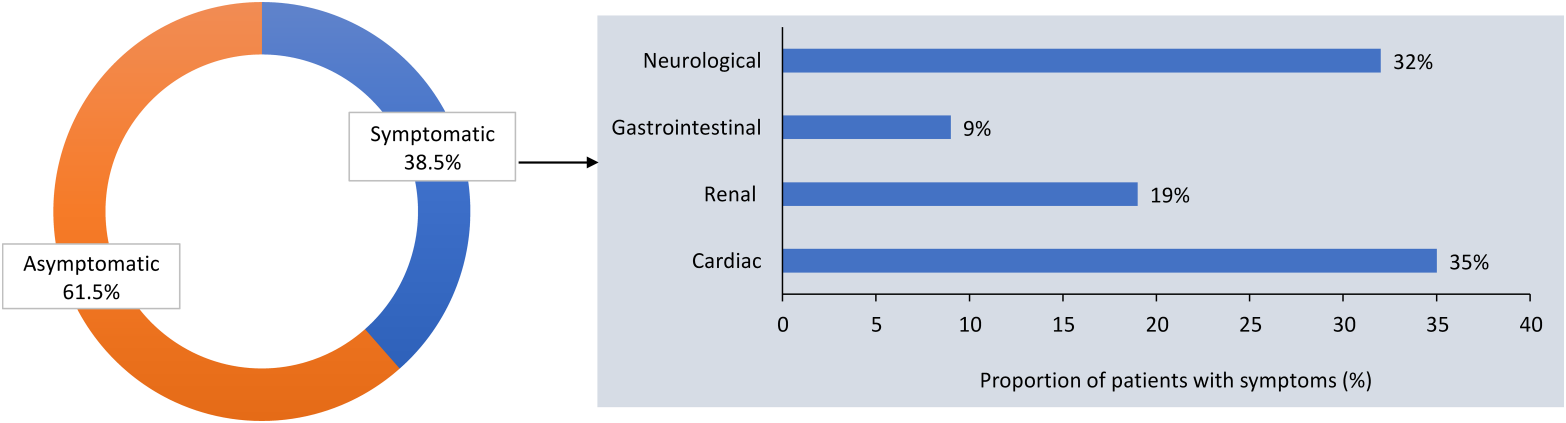
Proportion of asymptomatic and symptomatic females with Fabry disease at presentation, and clinical manifestations of Fabry disease in symptomatic females in the survey of Italian centers.

**Figure 2:**
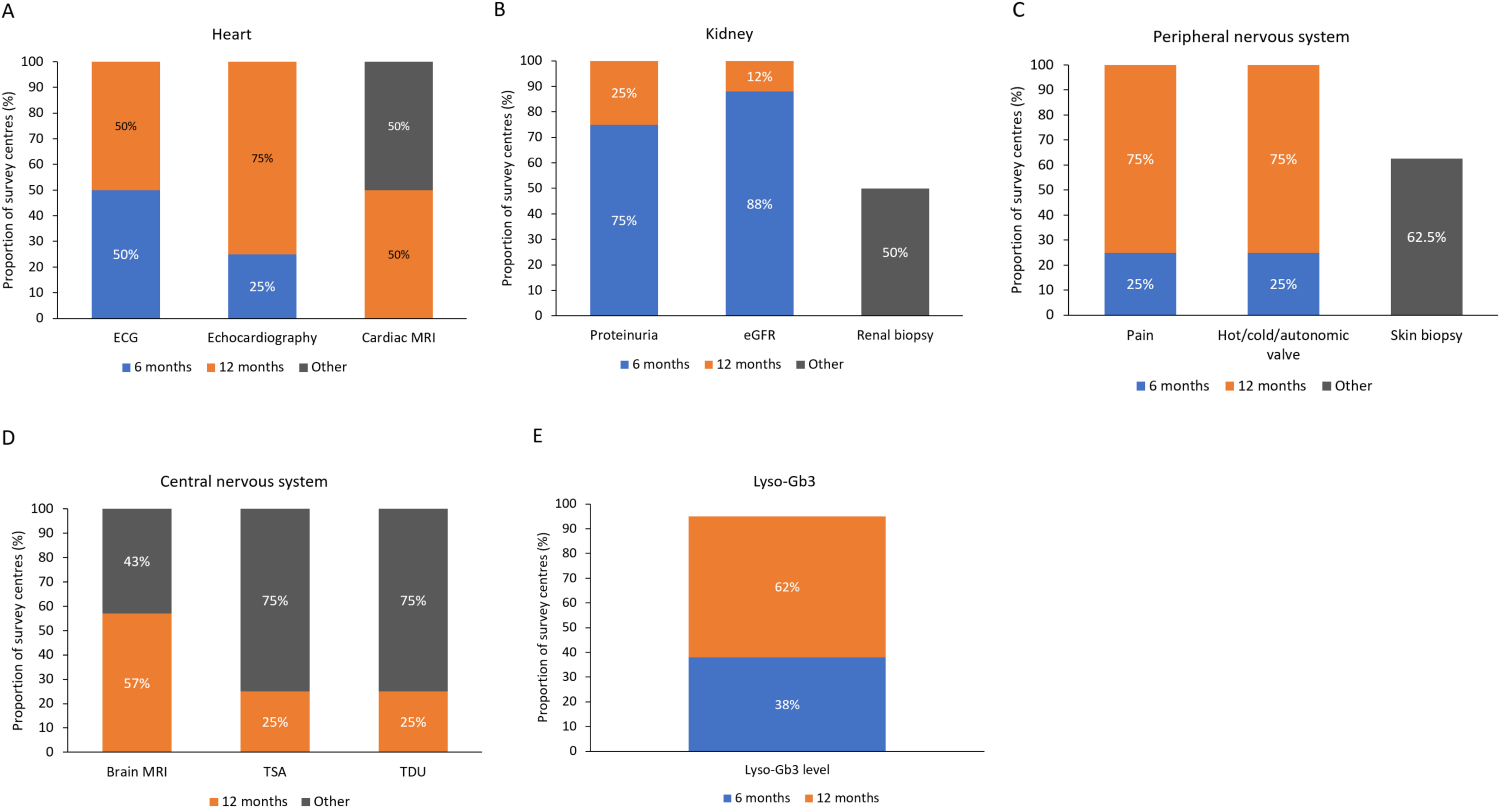
Monitoring of **(A)** heart, **(B)** kidney, **(C)** peripheral nervous system, **(D)** central nervous system, and **(E)** plasma lyso-Gb3 levels at different time intervals at the nine Italian centers participating in the survey. ECG, electrocardiogram; eGFR, estimated glomerular filtration rate; lyso-Gb3, globotriaosylsphingosine; MRI, magnetic resonance imaging; TDU, transcranial Doppler ultrasound; TSA, transcortical sensory aphasia.

According to the survey, 38.5% of the 227 female patients with FD were symptomatic at presentation, and the most common manifestations were cardiac, neurologic, renal, and gastrointestinal (GI) symptoms (Figure 1). More participating centers performed cardiac monitoring at 12-month than at 6-month intervals (Figure 2A). An electrocardiogram (ECG), echocardiogram, and cardiac magnetic resonance imaging (MRI) were used to monitor the heart, depending on clinical need. Cardiac MRI was performed every 24 months in 50% of the centers. In contrast, renal function was more commonly monitored at 6-month than at 12-month intervals, using daily proteinuria and estimated glomerular filtration rate (eGFR) assessments (Figure 2B). In 50% of centers, renal biopsy was considered during monitoring, in the case of sudden renal function deterioration or onset of overt proteinuria. Involvement of the peripheral nervous system (PNS) was monitored by assessing pain, heat or cold sensitivity, and dysautonomic symptoms, mostly every 12 months (in 75% of the centers; Figure 2C). Skin biopsy was performed at 62.5% of the centers in selected patients.

Central nervous system (CNS) symptoms were monitored using brain MRI in 57% of the centers, and echo-doppler examinations of the supra-aortic trunks (DESAT) or transcranial doppler ultrasound (TDU) in 25% of centers, each at 12-month intervals, but not at 6 months (Figure 2D). Brain MRI was performed every 24 months in 43% of the centers. DESAT and TDU were also performed at diagnosis in 25% of centers each. Plasma lyso-Gb3 levels were monitored in 38% of centers at 6-month intervals and in 62% at 12- month intervals (Figure 2E).

**Figure 3:**
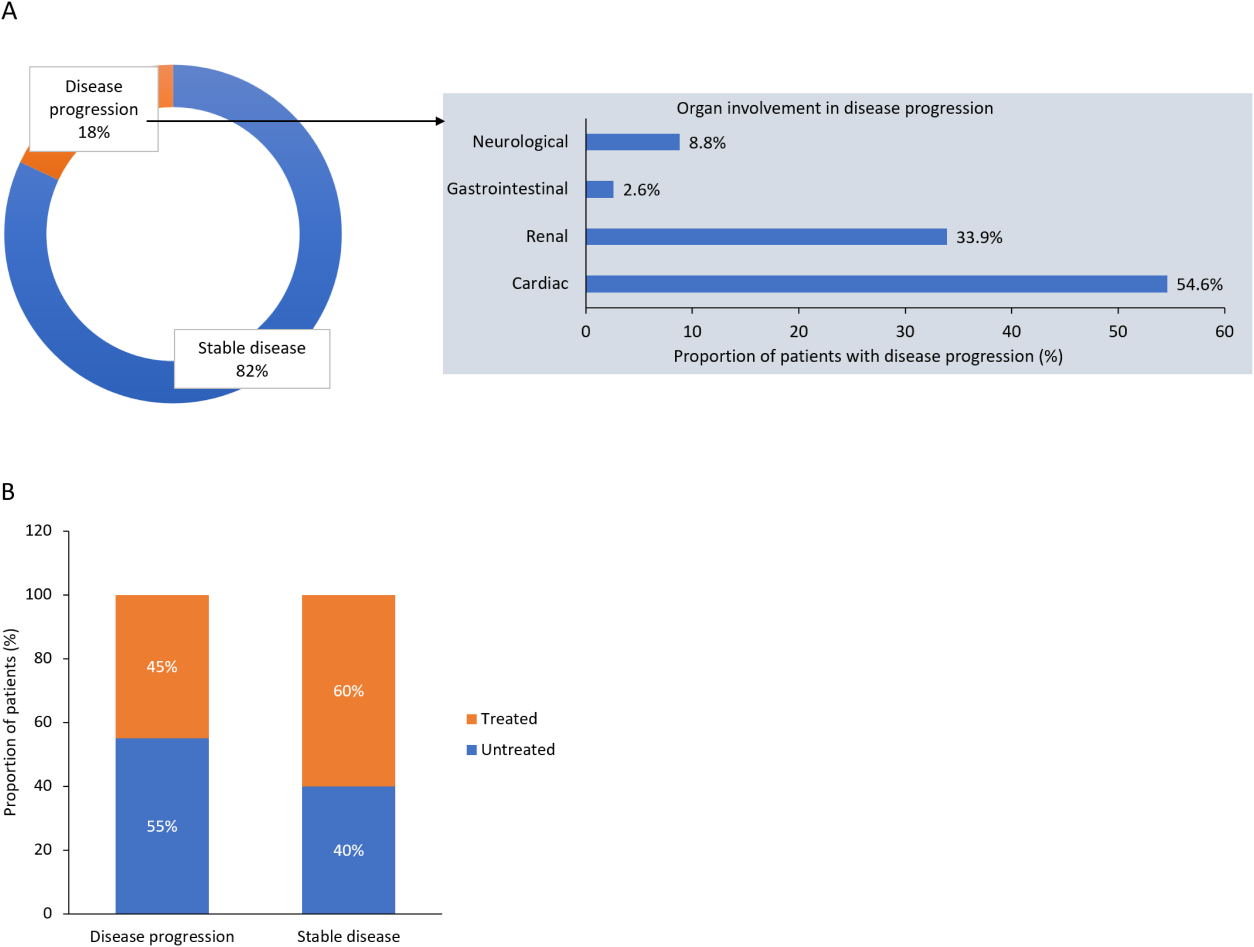
Disease status and organs involved in **(A)** disease progression, and **(B)** disease progression according to treatment status: results from the survey of Italian centers.

At the time of this survey, 82% of the female patients were in a stable condition, while 18% had disease progression (Figure 3A). In patients with progressive disease, the most commonly affected organs were the heart (in 55% of patients), kidney (34%), nervous system (9%) and GI tract (3%). Among patients who progressed, 45% were receiving treatment, while the remaining 55% were still without treatment. In patients with stable disease, 60% were receiving therapy and 40% were untreated (Figure 3B).

#### 3.2.2 Analysis

Life expectancy in untreated patients is approximately two decades longer in females with FD compared with males (36). The most common cause of death among both sexes is cardiovascular disease (36), with arrhythmias being the most frequent cardiac event (37). While approximately 50% of untreated male patients have cardiac rhythm disturbance by the age of 45 years, these events also affect 20%–45% of female patients aged 45–65 years (37).

Males and females with FD have similar clinical manifestations and organ damage, but they differ in the frequency, intensity, and onset of symptoms (16). However, with recent advances in molecular techniques, family screening, and better clinical characterization, clinicians are able to detect the non-classical forms of FD, and importantly, able to diagnose more female patients.

Data indicate that males with classical FD have a much higher risk of developing cardiac, renal, or cerebral events than those with non-classical FD and female patients with either phenotype (2). Females with classical disease are also at higher risk of developing complications compared with those with non-classical FD; however, the difference in risk in females is lower than for male patients with classical vs. non-classical FD (2).

Cerebral small vessel disease is often observed in patients with FD. In a case-control study (43% of the total cohort was female), the prevalence of impaired cerebral autoregulation was assessed using TDU as a biomarker for cerebral small vessel disease, with the finding that impaired cerebral autoregulation was more prevalent in patients with FD than in healthy controls (38). However, the indices of this impairment did not show independent association with white matter hyperintensities; in addition, the indices had low-to-moderate predictive ability for discriminating FD patients with and without white matter hyperintensities.

Strategies for disease monitoring should include an assessment of pathologic, metabolic, and clinical phenotypes; however, this is not always practiced in female patients. There is currently a lack of data about symptoms specific to female patients. Typical symptoms of FD should be proactively investigated in females, who tend to ignore their symptoms, continuing to live with them for years before reporting them to their primary care physician.

Data suggest that more females than males experience pain in their hands and feet (39% vs. 29%) and abdomen (30% vs. 22%) (39, 40). The frequency and severity of diarrhea, the second most common GI symptom after abdominal pain, are more variable; 20% of FD patients report diarrhea, which is more common in males (26%) than females (17%) (41).

Renal biopsy can be a helpful diagnostic and monitoring tool for females, especially when there is uncertainty about the actual presence of the disease (42). Historically, renal biopsy was thought to be of little value in females because of the possible presence of mosaicism and VUS; however, it is now known that renal histology is similar in male and female patients, with frequent accumulation of Gb3 inclusions in affected renal podocytes. Tissue damage precedes the presentation of signs and symptoms and, therefore, the Gb3 inclusions may not necessarily be associated with proteinuria or renal dysfunction (43, 44). This accumulation of Gb3 and subsequent podocyte injury progresses with age, indicating that renal biopsy could help to identify females with a greater risk of CKD progression (45).

Evidence indicates that cardiac biopsy can also be useful in making a diagnosis. Cardiac symptoms in females are often misunderstood or neglected, even in the general population, but it is important to pay attention to the earliest signs of cardiac damage in females with FD to avoid progression to microvascular ischemia. FD patients often have structural myocardial changes and altered ECG parameters even before they have developed LVH or detectable sphingolipid storage on MRI (46).

The pathogenesis of cardiac damage may differ between males and females with FD. One study found that in males with FD, the development of hypertrophy was always followed by the development of myocardial fibrosis, whereas in females with FD, myocardial fibrosis sometimes occurred in the absence of hypertrophy (47). A study of 34 female patients with late-onset hypertrophic cardiomyopathy found that 12% had FD (48). The biopsies of females with FD showed patchy myocardial distribution of cells containing glycolipid inclusion vacuoles as clearly distinguishable islands of affected cells within areas of normal cells, whereas in males with FD, glycolipid-laden cells were diffusely distributed (48). Another study revealed that unaffected myocardial cells can also be abnormal, showing cell hypertrophy that correlated with the increase in left ventricular maximal wall thickness (49). This suggests that affected cells may have a negative paracrine effect on healthy neighboring cells, stimulating cellular hypertrophy that can contribute to the progression and severity of FD cardiomyopathy (49). Therefore, endomyocardial biopsy can provide valuable information for the diagnosis and treatment of FD in females, especially if VUS are present or when the only organ involved is the heart. The experts, therefore, suggest that cardiac biopsy should be considered in female patients with cardiac symptoms, even in the absence of LVH or other FD clinical manifestations, to clearly define myocardial involvement.

Skin biopsy may also be useful for the diagnosis of FD, although histologic skin changes tend to be less marked in female than male patients (50). Electron microscopic analysis of punch biopsy skin samples from patients with FD showed abnormal glycolipid storage in fibroblasts, but the number of affected fibroblasts differed between the sexes, with males showing an average of 90% affected and females an average of 15% (50). Similar differences between the sexes have been shown in skin biopsy samples analyzed using immunofluorescence; samples from patients with FD showed more Gb3 deposition compared with samples from healthy controls, but the magnitude of Gb3 deposition was greater in males than females with FD (51). Thus, cutaneous biopsy may be advisable in selected females with suspected FD and those with VUS.

The greatest decision-making issues for clinicians are choosing how often to monitor, and when to initiate therapy in, female patients with FD who are completely asymptomatic and identified by family screening or for reasons other than symptoms. For example, the unconventional progression of heart damage in females with FD raises the question of whether treatment should be started early or delayed until all biomarkers are identified. Early biomarkers of disease progression are crucial for treatment decision-making and in preventing organ damage in patients with actual risk of progression. The combination of MRI tissue mapping sequences and ECG appears promising to detect early myocardial changes (46), because currently it is not possible to use the development of hypertrophy or fibrosis as a biomarker for the prevention of cardiac damage.

Proteomics have been used to search for novel plasma biomarker signatures to improve disease prognosis; Hollander and colleagues reported sex-specific proteomic signatures (52), whereas L'Imperio and colleagues reported phenotype-specific proteomic signatures, but no sex-specific differences (53). This phenotypic-specific signature is a promising approach in helping the correct classification of FD phenotypes and interpreting undetermined mutations or VUS (53).

The role of single nucleotide polymorphisms (SNPs) in the monitoring of FD has historically been neglected, but SNPs could help in risk stratification, provide valuable information on prognosis and help personalize disease management. For example, an SNP upstream of the promoter of the *GAL* gene (−1° C→T) in patients with FD reduces the transcription of the gene and the residual expression of *α*-Gal A compared with the effect of a *GAL* mutation alone, and increases the patient's predisposition to have a transient ischemic attack (TIA) (54). While *GAL* mutations cause FD, SNPs increase the risk of developing organ damage during the disease course, but it is difficult to assess the impact of SNPs because of similarities between the effects of mutations and polymorphism. Therefore, SNPs should be considered in association with *GAL* mutations. Neurologic manifestations can occur in patients with FD (55) and are characterized by stabbing pain and burning paresthesias in the extremities that are triggered by temperature changes and are often severe. Unfortunately, standard neurophysiologic procedures are inadequate to accurately assess the peripheral and autonomic nervous systems of FD patients with these symptoms. Alternative methods to determine the extent of neurologic dysfunction have been developed and include assessment of impaired temperature perception, vibratory perception, sudomotor and sweat gland function, blood flow, and vasoreactivity of the limbs and superficial skin using thermal provocation tests, the quantitative sudomotor axon reflex test, and venous occlusion plethysmography (56).

Peripheral neuropathic manifestations present later in females than in males (39, 57), but the burden of neurologic disability increases with age in both sexes (58). Targeted neurologic tests at 20–30 years of age, before Gb3 accumulation in renal or cardiac cells, could allow for earlier initiation of therapy, with the hope of preventing the disease from worsening. The experts also suggested that assessing anhidrosis may be specifically useful, because this sign of autonomic function changes after starting therapy (i.e., patients with FD start sweating after starting treatment) (59), and can be easily performed in all clinics. There are scales to measure autonomous dysfunction, such as the Scales for Outcomes in Parkinson's disease (SCOPA) score (60), but such specific assessment scales are generally used only at second-level centers. These targeted neurologic tests should also be performed in patients with certain types of VUS that have been reported to be linked to peripheral neuropathic pathology (61) or in patients with VUS and peripheral neurologic symptoms.

Assessment of α-Gal A activity in females with suspected FD is not useful for diagnostic purposes, but may help to assess disease progression when used in conjunction with plasma lyso-Gb3 levels (62, 63). It is important to remember that, while increased plasma lyso-Gb3 levels are suggestive of FD, normal levels cannot exclude the disease, especially in females with VUS (21). In asymptomatic females with VUS and no family history of FD, histologic evaluation of the kidney or lyso-Gb3 levels may aid in treatment initiation decision making (64). In female patients with classic FD, Gb3 accumulation could also occur in renal and cardiac cells in childhood (65, 66).

The attending physician should actively investigate all signs and symptoms, but unfortunately, in clinical practice, investigation of medical history tends to vary depending on the specialty of the physician. It is, however, difficult for any specialist to link symptoms/events in a specific organ with the occurrence of a rare disease. For example, GI symptoms in FD are nonspecific and may be misdiagnosed as irritable bowel syndrome or colitis. There is still uncertainty about the best way to monitor female patients with FD (17), and there is no evidence that one approach is better than another. There is also no evidence that monitoring progression in females using the same tests and controls as are used in male patients is useful/necessary or whether it may be preferable to use assessments based on sex and disease phenotype.

A further challenge in monitoring asymptomatic female patients with FD is that they are often lost to follow-up due to lack of clinical manifestations. However, a long period without clinical manifestations does not rule out significant organ damage (e.g., fibrosis) or high risk of acute symptoms (e.g., stroke).

### 3.3 Treatment initiation and therapeutic efficacy in females

#### 3.3.1 Survey findings

**Figure 4:**
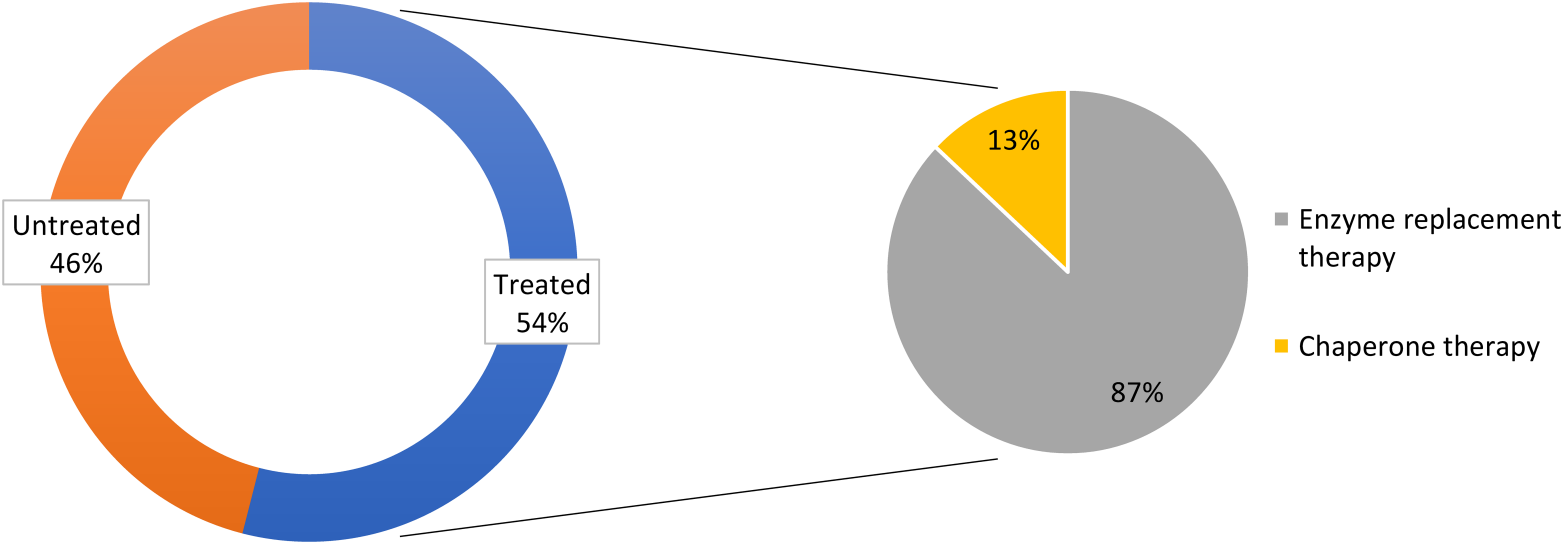
Treatment status of females with Fabry disease in the survey of Italian centers.

All survey centers used the following parameters for treatment initiation: plasma lyso-Gb3 levels in asymptomatic patients; signs and symptoms of damage to the heart, kidney, CNS, PNS, or GI tract; family screening; and *GLA* mutation genotype/pathogenicity. Among the 227 patients of the physicians surveyed, 54% were receiving therapy; of these patients, 87% were using ERT and 13% were treated with chaperone therapy (Figure 4).

#### 3.3.2 Analysis

Treatment decisions should be undertaken by a multidisciplinary team. The experts agreed that all clinical criteria for treatment initiation should be taken into account because, when considered individually, the only relevant criterion is the finding of signs and symptoms of FD, while evidence from family screening, Gb3/lyso-Gb3 accumulation or the presence of *GLA* mutation alone is not sufficient to initiate treatment. The genotype is related to the pathogenicity of the *GLA* mutation, for example, if there is a mutation in the stop-codon, Gb3 and lyso-Gb3 levels will increase and their accumulation will occur, which as per recommendations is sufficient to initiate treatment (17).

There is evidence that ERT is underused in females, even in symptomatic cases. According to the 2008 Fabry Registry Annual Report, at least 63% of females with FD did not receive ERT (16). In a multicenter clinical survey in Germany including 224 female patients with FD, approximately 34% of female patients did not receive ERT, despite fulfilling the criteria for ERT initiation (67). Among those who did receive ERT, 42% had two or three different manifestations (i.e., renal, cardiac, neurologic, or GI) and all of them had missense or nonsense *GLA* mutations (67). The Spanish Fabry women study reported that 57% of female patients did not receive treatment despite major organ involvement (15).

The experts agreed that a considerable proportion of female patients with stable disease do not receive treatment. However, they were unable to explain the reason why 46% of female patients in this survey were not receiving treatment, despite clear signs of disease burden, as this contrasted with available literature showing that therapy is effective in females (7). The low rate of treatment in females with FD reflects the fact there is often a delay in starting treatment either because they are asymptomatic and believed to be at low risk of progression or because patients themselves refuse the proposed therapy, especially if they are asymptomatic or paucisymptomatic. However, several reports have documented a high risk of events in untreated females (68) or better cardiac and renal outcomes in females after the beginning of treatment than in the pre-treatment period (69).

The effects of therapy depend on the disease stage at treatment initiation (70); if treatment is initiated at a later stage when irreversible organ damage has already occurred (e.g., the patient has renal failure and is already on dialysis, or the patient has advanced hypertrophic cardiomyopathy), therapy may not be able to stabilize disease progression in the associated organ systems. The experts recommended that treatment in females should be started based on the age of the patient, *GLA* mutations, pathogenicity, and signs and symptoms of FD. It was previously recommended that treatment in females should only be initiated if there are significant symptoms or there is evidence of progression of organ damage, such as chronic peripheral neuropathic pain resistant to conventional therapies, persistent proteinuria (300 mg/24 h), eGFR <80 ml/min/1.73 m^2^, clinically significant cardiac involvement, history of brain stroke, or TIA or ischemic changes on brain MRI (71). However, survival data showed a lower life expectancy in untreated females with FD compared with the general female population (2), leading to a change in treatment approach.

According to the revised recommendations, ERT can also be initiated in asymptomatic females on the basis of laboratory parameters (e.g., eGFR <90 ml/min/1.73 m^2^; albuminuria >30 mg/dl), histologic findings (e.g., renal Gb3 inclusions), imaging (e.g., silent strokes or cerebral white matter lesions on brain MRI; cardiac fibrosis on contrast cardiac MRI), or molecular analysis (e.g., skewed XCI pattern with predominant expression of the mutant *GLA* allele) (17). In the authors’ opinion, increasing levels of lyso-Gb3 should be considered when deciding whether to initiate treatment in asymptomatic females. Moreover, in patients with late-onset FD, ERT should be considered if there is evidence of injury to the kidney, heart, or the CNS (as detailed above), even if there are no typical FD symptoms. Such abnormalities should be attributable to FD and require histologic assessment or biochemical evidence of Gb3 accumulation (17).

There are several data that support the efficacy of ERT (agalsidase-β and agalsidase-α) and chaperone therapy (migalastat) in female patients (69, 72–74). Agalsidase-β was particularly effective in stabilizing left ventricular posterior wall thickness, interventricular septal thickness, and eGFR decline in females (69). Moreover, females who were treated with agalsidase-β for up to 2 years experienced significant improvements in various aspects of health-related quality of life, including body pain, vitality, physical function, general health perception, mental health, and social function (73). Similarly, the long-term effectiveness of agalsidase-α has been demonstrated in females with FD, with reductions in the severity of disease (as measured by the Mainz Severity Score Index) and left ventricular mass (LVM), an improvement in New York Heart Association heart failure classification, and stabilization of kidney function over 4 years of treatment (72). In a phase 3 study of migalastat vs. ERT, in which 56% of the study cohort were female, after 18 months, the treatments had similar effects on renal function, renal/cardiac/cerebrovascular events and patient-reported outcomes (including assessment of pain), but migalastat induced a greater decrease of LVM (74).

There are limited data comparing ERT tolerability between males and females, but females may tolerate ERT better because they are less likely to develop anti-drug antibodies than male patients (75).

## 4 Expert Opinion

### 4.1 Recommendations for current practice

Diagnosis, monitoring, and management of FD in females has been an area of concern for many years (76). The recommendations from this expert group are summarized in Table 1.

**Table 1:** Expert panel recommendations on the diagnosis, monitoring, and initiation of treatment for female patients with Fabry disease.

Diagnosis and screening
• Screen all at-risk female relatives (identified by pedigree creation) of an index case to minimize the number of patients going undiagnosed
• Genetic analysis is the only reliable method for FD diagnosis in female patients
• Do not use α-Gal A activity and plasma lyso-Gb3 levels for diagnosis of FD in females as these parameters have low diagnostic sensitivity in females
• XCI analysis can be a useful adjunct to guide monitoring and treatment decisions
• Consider all organ systems that may be affected (e.g., heart, kidney, PNS, CNS and GI tract) and evaluate the global clinical picture
• In older patients with late-onset diagnosis, evaluate whether clinical manifestations are related to FD or aging
• Organ biopsy (renal or cardiac), and/or cutaneous biopsy, should be considered in selected asymptomatic patients and in those with *GLA* VUS, to assess histological organ involvement
• Biopsy can be avoided for FD diagnosis in patients with classical FD because the results are predictable, but they can be useful during follow up to assess disease progression despite treatment
• Cutaneous biopsy may be advisable in patients with suspected FD and those with VUS
Monitoring
The monitoring schedule for asymptomatic females must balance the need to detect organ damage with the need to maintain adherence (asymptomatic females are often lost to follow-up)
• Assess renal function annually using daily proteinuria and eGFR
• Begin peripheral neurologic testing every 2 or 5 years at 20–30 years of age to detect early changes
• One-off assessment of anhidrosis is an easy-to-measure parameter of autonomic dysfunction
• Annual CNS monitoring using MRI and cognitive function tests is useful
• Begin annual echocardiographic monitoring from 35 years of age because signs of damage appear after age 45 years
• The role of α-Gal A activity and plasma lyso-Gb3 levels on disease progression has not been confirmed
Treatment initiation
• The decision on when to initiate therapy in females should be based primarily on specific signs and symptoms of FD (see Diagnosis and screening)
• Treatment can also be initiated in asymptomatic patients taking into account their age, results of family screening, *GLA* mutations, Gb3/lyso-Gb3 accumulation, and histologic or other evidence of organ damage (in the absence of overt symptoms)

α-Gal A, α-galactosidase A; CNS, central nervous system; eGFR, estimated glomerular filtration rate; FD, Fabry disease; Gb3, globotriaosylsphingosine; *GLA*, alpha-galactosidase A gene; GI, gastrointestinal; lyso-Gb3, lyso-globotriaosylsphingosine; MRI, magnetic resonance imaging; PNS, peripheral nervous system; VUS, variant of unknown significance; XCI, X chromosome inactivation.

Regarding the diagnosis of females with FD, the experts noted that FD indeed causes less obvious symptoms in females, but also that the clinical manifestations of FD in females are less explored. It is important to ensure that all at-risk female relatives of an index case are screened for FD, to minimize the number of cases remaining undiagnosed. At-risk females may be identified by building a genealogical tree of mutations (i.e., pedigree creation). As FD is a multisystemic disease, to accurately diagnose FD, it is important to consider all of the organ systems that may be affected (e.g., heart, kidney, nervous system, and GI tract), rather than limiting the evaluation to one particular organ. It is also important to remember that symptoms in the different systems, particularly in the nervous system, may be blurred, and that diagnosis can only be achieved by carefully examining the global clinical picture. In the case of a late-onset diagnosis, it is critical to evaluate whether the clinical manifestations are related to FD or aging.

Although genetic analysis is the only reliable method for FD diagnosis in females, many laboratories outside of Italy still rely on the less sensitive measurements of α-Gal A activity and plasma lyso-Gb3 levels. The experts strongly suggest that laboratories should discontinue the use of these less sensitive methods for FD diagnosis. Although no epigenetic evaluation was reported in this survey, the XCI profile (when assessed) may guide treatment decisions in female patients with FD (17).

The experts suggested that it is important to pay attention to the earliest signs of organ damage to avoid disease progression. Females tend to ignore their symptoms, while at the same time their symptoms are often misunderstood or not given the attention they deserve by clinicians. Because organ damage is common, even in the absence of symptoms, a biopsy may be extremely useful for diagnostic and therapeutic decision making and monitoring purposes, even outside the research setting. Thus, the experts recommend that organ biopsy (either renal or cardiac) and/or cutaneous biopsy (a less invasive diagnostic tool) should be considered in female patients, in particular in those who are young, asymptomatic, and with *GLA* VUS. Biopsy may be avoided, however, in patients with classical FD as the outcome is predictable.

A follow-up approach based on age should be implemented, considering the increased risk of certain manifestations at different times of life. For example, echocardiographic parameters are normal until the ages of 35–44 years in female patients with N215S *GLA* mutation, but signs of damage appear after the age of 45 years (77). It is important to take care when monitoring young patients, as they may tire of repeated negative outcomes and may not be as willing to participate in ongoing follow-up.

The experts also suggest that it may be helpful to consider neurologic/psychologic symptoms (e.g., depression), which tend to be neglected in females, or neurosensitive symptoms, which are often misinterpreted and lead patients to consult different specialists and may sometimes result in an incorrect diagnosis. Females with neurosensitive symptoms or pain without cause are often diagnosed as having fibromyalgia. In addition, more FD centers should adopt brain MRI for CNS involvement.

The experts agreed that the Italian approach to treatment initiation has been conservative, but they believe that while symptomatic females should be immediately started on therapy, asymptomatic females should be carefully monitored, and treatment initiated at the first signal of change. However, in the absence of symptoms, plasma Gb3/lyso-Gb3 levels, presence of missense or nonsense *GLA* mutation, and family history should be considered when initiating treatment, especially in younger patients. In female patients with VUS, the decision to start treatment may be considered only if there is evidence of pathogenicity.

### 4.2 Recommendations for future research

Despite recent progress, many unanswered questions remain about the assessment and management of female patients with FD. It will be important to specifically design studies to identify new sensitive and reliable biomarkers for the diagnosis and monitoring of FD in female patients. Knowledge gaps exist in the following areas: the diagnostic role and impact of XCI; the role of XCI in the decision to start therapy early; the use of biomarkers for diagnosis; the mechanisms underlying organ damage in FD; understanding the pathogenesis of Gb3/lyso-Gb3 accumulation and basic cellular pathology; identification of prognostic markers to establish the most appropriate time for treatment initiation, especially in asymptomatic or paucisymptomatic patients; the natural course of the disease in females with classical and non-classical FD; and the effect of therapy on non-classical female cases.

XCI evaluation depends on the following: extensive assessment of DNA methylation patterns of CpG islands of X chromosomes; the choice of tissue, as the mosaicism is tissue-dependent and may result in different test outcomes; and the use of uniform methodologies so that studies can be compared. However, in patients with large *GLA* mutation variabilities, including VUS, it would be difficult to find correlations between XCI and phenotype because of upstream bias.

XCI may affect the phenotype but the severity of the *GLA* mutation prevails over XCI; therefore, it is essential to study patients with homogeneous *GLA* mutations to identify a reliable biomarker for genotype-phenotype correlation. Table 2 lists recommendations for the steps needed to identify a predictive marker.

**Table 2:** Expert panel recommendations for the assessment of predictive epigenetic markers.

• Consider testing for predictive epigenetic markers in large families
• Consider genes close to *GLA*, as their interaction can define the target organ and also influences disease expression in the family setting
• After establishing the haplotypic structure of individual genes within the family, study each epiallele using integrated whole-exome and mRNA sequencing
• Verify the marker in four reference tissues that can be easily collected, such as blood, saliva, urinary exosomes, and skin biopsy, and in all identified families to assess the tissue-specific variability
• Statistically validate the marker

*GLA*, alpha-galactosidase A gene.

The experts noted that it would be interesting to study organ-specific lyso-Gb3 isoforms as this may explain the variability of disease phenotype within the same family carrying the same mutation. Currently, some data may be available in animal models, but not in humans or organ biopsy samples.

The data on untreated females must be reviewed to understand whether the failure to receive treatment depends on the actual absence of symptoms, patients' resistance to treatment, clinicians' therapeutic inertia, or underestimation of symptoms, or on a difference in attitude towards the treatment of male and female patients with FD. There is also a need to dissipate the perception that heterozygous female carriers have less severe disease and are not eligible for treatment unless they are symptomatic. ERT should be proposed with equal conviction in male and female patients and should also be based on molecular testing/genetic diagnosis and not only on phenotype.

Additional therapeutic agents are being studied, including substrate reduction therapies such as lucerastat and venglustat. Gene therapy has also been assessed in both animal models and early-phase clinical trials, with initial results indicating favorable tolerability and efficacy. Given the activity in this area, it is likely that more therapeutic options will become available in the near future, which should provide clinicians with the ability to offer patients a more individualized approach to the management of FD (78).

There is a need to have more data on FD treatment in females to address uncertainties about disease evolution in treated vs. untreated females. Such knowledge may help to maximize adherence to treatment in female patients, especially if they are uncertain of the benefit.

It is important to aim for personalized therapy in females. To do so, a genealogical tree of GLA mutations should be created to trace patterns of disease transmission and predict the possible clinical implications for the patient, as this information influences the choice of treatment. Notably, the phenotype-genotype correlation is not constant within a family. Decision-making algorithms based on the patient's genetic profile and polymorphisms will allow clinicians to decide who to treat, irrespective of symptoms or organ damage.

Lastly, key messages about clinical management of female patients with FD need to be widely and effectively disseminated, with more conventions, investigator meetings, and educational meetings on FD in females to increase awareness among clinicians.

## 5 Conclusions

Many challenges and unmet needs exist regarding the management of FD in females, with evidence indicating that females are at a significant disadvantage compared to males with FD in terms of timeliness of diagnosis and initiation of treatment. More research into FD in females is needed and greater physician awareness is required to improve outcomes in this underserved patient population.
