# Supplementary material for: Females with Fabry disease: an expert opinion on diagnosis, clinical management, current challenges and unmet needs
Source: Front Cardiovasc Med. 2025 Mar 12;12:1536114. doi: 10.3389/fcvm.2025.1536114 (PMC11937019; doi:10.3389/fcvm.2025.1536114)
Supplement: Supplementary file 1 [file Supplementaryfile1.pdf]

Supplementary Material

Physician survey regarding characteristics and management of female patients with Fabry disease

1. Indicate the number of female patients followed at your site.

2. Of these, please indicate the percentage of women who are diagnosed.

- Through family screening
- Through disease manifestations (proband)

3. Indicate the workflow mainly followed for diagnosis in women.

- Enzyme dosage followed, in the case of reduced activity, by molecular investigation
- Direct molecular investigation
- Enzymatic assay + globotriaosylsphingosine (lyso-Gb3) followed, in case of reduced enzyme activity and/or increase in lyso-Gb3, by molecular investigation

4. Indicate whether and what percentage of the epigenetic assessment (X chromosome inactivation) is performed, as a predictive marker of disease evolution (if any, indicate the laboratory).

- Yes (%)
- No (%)

5. Indicate, in the female population followed at the site, what the manifestations were at onset (in percentage).

- Cardiac (%)
- Renal (%)
- Gastrointestinal (%)
- Neurological (%)

6. Indicate, in the female population followed at the site, what was the disease progression.

- No progression (%)
- Of these, how many patients were:
    - Treated (%)
    - Untreated (%)
- Progression (%)
- Of these, how many patients were:
    - Treated (%)
    - Untreated (%)
- Indicate, as a percentage, the type of progression:
    - Cardiac (%)
    - Renal (%)
    - Gastrointestinal (%)
    - Neurological (%)

7. Indicate how disease follow-up is performed.

- Visit frequency:
    - 6 months
    - 12 months
    - Other
- Frequency of clinical and instrumental tests:
    - Heart:
        - Electrocardiogram (ECG): 6 months/12 months/other
        - Echocardiogram (ECHO): 6 months/12 months/other
        - Cardiac magnetic resonance imaging (MRI): 6 months/12 months/other
    - Renal function:
        - Proteinuria: 6 months/12 months/other
        - Estimated glomerular filtration rate (eGFR): 6 months/12 months/other
        - Biopsy: 6 months/12 months/other
    - Peripheral nervous system (PNS):
        - Pain assessment (Neuropathic Pain Symptom Inventory [NPSI] and Brief Pain Inventory [BPI]): 6 months/12 months/other
        - Hot/cold intolerance, sensory testing and autonomic assessment: 6 months/12 months/other
        - Skin biopsy: 6 months/12 months/other
    - Central nervous system (CNS):
        - Echo-doppler examinations of the supra-aortic trunks (DESAT): 6 months/12 months/other
        - Transcranial Doppler ultrasound (TDU): 6 months/12 months/other
        - MRI: 6 months/12 months/other
    - Lyso-Gb3: 6 months/12 months/other

8. What are the parameters that are taken into account for treatment initiation (indicate with an X)?

- Lyso-Gb3 increase in asymptomatic patient
- Signs and symptoms of organ damage:
    - Cardiac
    - Kidney
    - Central nervous system (CNS)
    - Peripheral nervous system (PNS)
    - Gastrointestinal
- Familiarity
- Mutation genotype/pathogenicity
- Other

9. Please indicate the percentage of women currently being treated.

- Of these, please indicate the percentage of women receiving:
    - Enzyme replacement therapy (ERT)
    - Oral therapy
